# A Corpus Investigation of Syntactic Embedding in Pirahã

**DOI:** 10.1371/journal.pone.0145289

**Published:** 2016-03-02

**Authors:** Richard Futrell, Laura Stearns, Daniel L. Everett, Steven T. Piantadosi, Edward Gibson

**Affiliations:** 1 Department of Brain and Cognitive Sciences, Massachusetts Institute of Technology, Cambridge, MA, United States of America; 2 Dean of Arts and Sciences, Bentley University, Waltham, MA, United States of America; 3 Department of Brain and Cognitive Sciences, University of Rochester, Rochester, NY, United States of America; Stony Brook University, UNITED STATES

## Abstract

The Pirahã language has been at the center of recent debates in linguistics, in large part because it is claimed not to exhibit *recursion*, a purported universal of human language. Here, we present an analysis of a novel corpus of natural Pirahã speech that was originally collected by Dan Everett and Steve Sheldon. We make the corpus freely available for further research. In the corpus, Pirahã sentences have been shallowly parsed and given morpheme-aligned English translations. We use the corpus to investigate the formal complexity of Pirahã syntax by searching for evidence of syntactic embedding. In particular, we search for sentences which could be analyzed as containing center-embedding, sentential complements, adverbials, complementizers, embedded possessors, conjunction or disjunction. We do not find unambiguous evidence for recursive embedding of sentences or noun phrases in the corpus. We find that the corpus is plausibly consistent with an analysis of Pirahã as a regular language, although this is not the only plausible analysis.

## Introduction

One of the most important empirical programs in cognitive science and linguistics aims to characterize the range of possible human languages. Linguistic universals—if any exist (see [1, 2])—would point to deep properties of the cognitive mechanisms supporting language; at the same time, the search for possible universals and violations of universals creates rich data for linguistic theory.

To date, one of the most compelling hypothesized universals is *recursion*, a computational mechanism that is central to modern linguistics, yet is frequently discussed with considerable terminological and conceptual sloppiness (see [3, 4]). Hauser, Chomsky & Fitch [5] (henceforth HCF) argue that recursion is *the* unique and defining feature of human language, contrasting the rich productivity and structure observed in human language with the relatively restricted systems of animal communication. HCF do not define the term, instead giving only the example of sentential embedding: “There is no longest sentence (any candidate sentence can be trumped by, for example, embedding it in ‘Mary thinks that …’), and there is no non-arbitrary upper bound to sentence length.” (p. 1571) (For detailed discussions of this and related issues, see [6–8]).

In contrast, Everett [9] argued that the Pirahã language lacked such embedding and indeed has an upper-bounded sentence length. In response to Everett, Nevins et al.[10] argued that the key sense of recursion relevant to HCF is instead that of repeated application of a binary structure-building operation, *Merge* (for more on this debate, see [11, 12]). Any sentence with more than two words (e.g. “Bill played accordion.”) is recursive in this sense because it has re-applied *Merge* to its own output in order to derive the full sentence structure via the formal tools of Minimalism. As Nevins et al.[10] write, “Hauser, Chomsky, and Fitch (2002, HC&F) presupposed, rightly or wrongly, an approach to syntactic structure in which all phrase structure—not just clausal embedding or possessor recursion—serves as a demonstration of recursion. We had this in mind when we noted in NP&R that if Pirahã really were a language whose fundamental rule is a non-recursive variant of Merge, no sentence in Pirahã could contain more than two words.” Pirahã clearly meets this narrow sense of recursion because it does have sentences longer than two words.

In our view, much of this prior debate is over essentially terminological issues which are orthogonal to an important and fascinating empirical program of characterizing what structures are present and absent in human language very broadly. Here, we aim to move past the “recursion debate” and provide data relevant to characterizing the complexity of Pirahã in terms of well-established concepts from formal language theory [13]. The conception of syntax here is purely about the combinatorial properties of well-formed sentences, without regard to semantic or discourse relations, which may have recursive structure of their own [14]. In this work, in order to clearly circumscribe our goals, we take semantic and discourse structure to be conceptually independent of syntactic structure, as in [15, 16].

In order to study the formal complexity of Pirahã and to provide data to inform the debate, we compiled, annotated, parsed, and analyzed a novel corpus of natural Pirahã consisting of stories which were originally collected and translated by Steve Sheldon and Dan Everett across several decades. Our corpus provides a machine-readable, aligned translation between Pirahã and English, including shallow syntactic parses and approximate English glosses. The analysis we provide *is intended to be preliminary* rather than definitive, as we believe that formal tools which do not yet exist will be required to definitively answer questions about the computational complexity required for Pirahã grammar. In this paper we describe the corpus and discuss a number of examples from the corpus which are relevant to the formal complexity of Pirahã.

In our discussion we tentatively address two questions: (i) Does Pirahã grammar allow recursive embedding? (ii) Can Pirahã be reasonably analyzed as a regular language? By recursive embedding, we refer specifically to the ability of one linguistic unit (e.g. syntactic phrase) to contain units of the same type. In the language of rewriting grammars, this corresponds to the ability of one nonterminal type to be rewritten (perhaps through any sequence of derivations) to itself. Examples in English, according to a standard analysis, include coordinated phrases (“John sang and danced” contains a VP “sang and danced” composed of VPs “sang” and “danced”), nested possessives (“John’s mother’s father” is a NP consisting of NPs), and embedded sentences (“If Jack fell down the hill, then Jill would be sad”, a sentence containing other sentences). We aim to state this property in as theory-neutral form as possible in order to avoid committing to any framework within the class of approaches that posit hierarchical structure. The question of recursive embedding is relevant to whether a language contains an infinite number of sentences: if a grammar has embedding, then it can generate an infinite number of sentences of unbounded length. To preview our results, we find no unambiguous evidence of recursive embedding. The corpus is consistent with a regular grammar, though we cannot claim that this is the “best” grammar.

Research on the formal properties of natural languages has tended to explore upper bounds on their complexity. For example, while most languages appear to be mildly context sensitive [17, 18], a debate exists as to whether certain languages such as Old Georgian and Yoruba are even more complex [19–21]. Our data joins work on the *lower* bounds of the complexity of natural language. Given that variation in formal complexity exists, it would not be surprising if there existed languages with low formal complexity, such as regular or even subregular languages. Gil [22, 23] has discussed an analysis of Riau Indonesian as lacking hierarchical structure, and Jackendoff & Wittenberg [16] have discussed the lower bounds of complexity in terms of the relation between syntax and semantics. Kornai [24] argues that lower complexity in languages does not necessarily result in lower information carrying capacity.

We emphasize that the question of language complexity cannot be fully answered by any corpus study or set of example sentences. Any finite corpus or set of examples can be given a description as a finite language in principle. Corpus results—and example sentences—however, are still highly relevant to the question since they may *suggest* phenomena that are most naturally captured with one kind of grammatical structure rather than another.

Our first way of thinking about how to decide whether Pirahã—or any other language—permits embedding was to consider applying machine learning and computational techniques. In principle, all that must be done to determine if embedding is part of the best description of Pirahã is to compare grammars with and without embedding and see which best match the corpus. In the context of Bayesian model comparison, we could ask which grammar assigned the data highest marginal likelihood, perhaps even integrating out the grammar’s production probabilities. Alternatively, if we had effective techniques to infer grammars from data, we could apply them to the corpus and see whether the inferred grammar tended to allow embedding or not. These approaches draw on principles like Minimum Description Length [25] as a part of philosophy of science: the best scientific theory is that which most effectively and parsimoniously compresses the available data. This idea is extremely powerful and provides an elegant solution to the logical problem of language acquisition [26], which is closely related to the problem of grammar comparison faced here. Once the problem is specified to the required degree of precision, the logical tools for determining what theory is best are, in principle, fairly well-accepted.

The challenge, though, in this case is that in our view the quantitative tools are not up to the task. There are too many possible grammars to compare all of them (at least with current, known techniques), and inferring grammars from data is an extremely difficult inferential task that doesn’t appear to work very well, even in simpler cases like inference of morphological rules [27, 28]. We did initially explore algorithms for fitting probabilities in several fixed, hand-specified PCFGs, which is a well-understood, solved problem in natural language processing. The harder problem of model comparison has no effective solutions at this point, although advances in computational techniques will likely make this problem tractable—or brute-forceable—eventually.

However, we also realized that a formal comparison of grammars, while quantitative and objective in some senses, also obscures the most important parts of the data. Likely, only some portion of the data is relevant to the existence of embedding, and any formal model comparison will hinge critically on how it handles these few data points.

We therefore focus here on bringing out the data points that we believe will be most informative for any kind of model comparison, formal or informal. We make the entire dataset available with the hope that future work will be able to provide a formal version (e.g. based in MDL or probability) of these arguments.

## Overview of Pirahã

The Pirahã are an indigenous hunter-gatherer group of about 800 people living in the Amazon rainforest. The language is a member of the Mura family. The Pirahã are almost entirely monolingual and show little interest in outside cultures. They have been studied for a number of rare aspects of their culture and language. For example, their language has no exact cardinal or ordinal numbers, leading to the study of their concepts of exact number [29, 30].

Fuller descriptions of Pirahã grammar can be found in [9, 31]. What follows is a brief overview of relevant facts for the present work.

Pirahã has one of the smallest known phoneme inventories, but has complex prosody [32–35]. The phonological segments of Pirahã are /i/, /a/, /u/, /p/, /t/, /k/, /h/, /s/, /b/, /g/, and /ʔ/. In the orthography we adopt for this paper, < *x* > represents the glottal stop and < *o* > represents /u/. The sound /s/ is usually absent from women’s speech; women use /h/ where men use /s/. /k/ is possibly not a segment of Pirahã at all, but a portmanteau realization of /hi/ and /hu/ in fast speech. The language has two tones, high and low. We indicate high tone in this paper using an acute accent. There are numerous tonal perturbation rules and allophonic rules in the language.

The basic word order in Pirahã is Subject-Object-Verb. The syntax encodes information structure, in that elements can appear before the subject or after the verb if they are topics. The language makes extensive use of clitics for subject and object. The clitics in Pirahã are shortened forms of pronouns and nouns, such as the words for ‘woman’ *xipoihii*, ‘manioc’ *xagaisi*, ‘meat’ *xisi*, and a few others. They can appear before nouns to indicate possession. The common clitics are *ti* for the first person, *gí* for the second person, and *hi* for the third person and also sometimes for the first person. Subject and object clitics are often repeated in multiple positions in a sentence [36]. For example, a typical sentence might have the form Subject NP—Subject clitic—Adverb—Subject clitic—Object NP—Verb. For discussion of similar phenomena in Tzeltal, see [37].

As described in [31], Pirahã verbs are complex. There are roughly ninety verb roots in the language and, in the analysis of [31], sixteen suffix classes. Many of the suffixes described in earlier work are broken into finer-grained forms in this paper, following Everett’s more recent analysis. Pirahã, like many other languages [38], encodes evidential markers in its verbal morphology as affixes: -*híai* ‘hearsay’; -*sibiga* ‘deduction’; -*ha* ‘complete certainty’; and -∅ (zero affix) ‘assumption of direct knowledge’. Pirahã nouns are non-inflected.

Pirahã makes use of appositive structures, where two coreferential NPs occur adjacently, as in the English sentence *My brother, a pilot, flew airplanes*. The prosody of apposition in Pirahã is initially described in [31] and further in [39]. We see ample evidence of apposition—or parentheticals—in Pirahã in this paper.

## Methods: Corpus creation and design

We obtained glossed transcriptions of 17 stories in Pirahã, consisting of a total of 1149 sentences and 6830 words in our analysis. 13 of the stories were collected by Steve Sheldon in the 1970s, and the remaining 4 stories were collected by Dan Everett over the period 1980–2009. Each story was told by a single speaker with no recorded interruptions. The stories were transcribed by Everett or Sheldon; audio recordings are only available for stories 2 and 3. According to Everett, the texts are fairly representative of how the Pirahã tell stories to one another.

In the initial format that we obtained, most of the texts included a sentence-by-sentence translation of the story in English (written by Sheldon or Everett), and a morpheme-by-morpheme transcription and gloss. In the corpus that we are making available, we have largely preserved the free translations, the Pirahã transcriptions, and their interlinear glosses. Some of the morphemes and word boundaries have been edited from the original for consistency and to reflect better translations according to Everett or Sheldon. An example of a word boundary adjustment involves separating the third-person clitic *hi* from the verb in many cases, which was erroneously included as part of the verb in the original transcriptions.

We have standardized the tone system to Everett’s two-tone system. In the machine-readable format we distribute, high tones are represented with uppercase vowels. In the text of this paper, we represent high tone with acute accents.

We attempted to break the texts up into sentences in a consistent way, adding sentence boundaries following Everett’s analysis. We consulted with Steve Sheldon in difficult cases. The resulting corpus has 1149 sentences, with an average length of 5.9 words per sentence. Many of the sentence breaks that we include were not included in the original transcriptions, due to changing analyses of the language. Our corpus also contains the sentence breaks as originally transcribed by Sheldon or Everett; following that analysis, the corpus has 745 sentences. The question of what constitutes a full sentence and what constitutes a sentence fragment is extremely subtle; these decisions were made according to Dan Everett’s judgment.

We added parts of speech to the morphemes and parsed the sentences shallowly, demarcating NPs and PPs and giving their grammatical relations. We used the following grammatical relations: subject, object, indirect object, locative, temporal, instrumental, vocative, topic. Without evidence for a distinct verb phrase, the sentence level category consists of a verb together with its dependent noun phrases or adpositional phrases.

The word order in Pirahã is predominantly verb-final, with subjects (S) usually preceding objects (O) for a predominantly SOV word order. Following Everett’s analysis, many noun phrases appearing outside this canonical order were labeled as topics. For example, if the subject intervened between the object and the verb (as in an OSV order), the object was labeled as a topic-obj. Similarly, noun phrases appearing after the verb were labeled as topics. Null subjects or verbs (indicated by *) were added to sentences which lacked a subject prior to the verb or a verb following the subject. Verb affix morphemes appearing without a verb morpheme were treated as evidence for a null verb.

We used labels similar to the Penn Treebank labels for syntactic categories [40]: *NP* (noun phrase); *IN* (adposition); *PP* (adpositional phrase); *VP* (verb phrase); *S* (sentence); *NN* (a common noun); *PRP* (pronoun); *NNP* (proper noun); *POS* (possessive NP); *JJ* (adjective); *DT* (determiner); *CD* (quantity term); *RB* (adverb); *FW* (foreign word); *FRAG* (fragment). We also introduced the symbol *Q* dominating the contents of direct speech reports.

The corpus is divided into stories; stories are divided into “utterances”; and utterances are divided into sentences. “Utterances” correspond roughly to the sentences delineated in the original transcription. Each sentence is given a unique numeric code, such as 11.14.3, meaning that it is a sentence from the 11th story, 14th “utterance”, 3rd sentence. Our corpus file is released as a simple machine-readable text file, and the shallow parses are compatible with tgrep2 searches. The text file also includes English glosses and is particularly convenient to search in English in order to find, for instance, all Pirahã sentences that have “and” in the English gloss.

We release the corpus under a Creative Commons Attribution-ShareAlike 4.0 International license, allowing for free use and re-use of the corpus so long as modifications and additions are distributed according to the same terms. The files are available as a repository in the Open Science Framework at http://osf.io/kt2e8 and also on GitHub at http://github.com/languageMIT/piraha.

## Phenomena of Interest

In this work we want to draw out the corpus examples of structures that have bearing on Pirahã’s formal language-theoretic characterization, i.e. whether the language is infinite or not, and whether the grammar that generates it is (sub)regular or context-free. We do not attempt to distinguish among the complexity classes of the subregular hierarchy here. Here we discuss the kinds of syntactic constructions we have searched for, and our motivation for searching for them.

We were primarily interested in evidence of *syntactic embedding*, where a constituent is embedded in another constituent of the same type. If this process exists and can be iterated without bound, then sentences of arbitrary length are allowed, and the language is infinite. While searching for cases of embedding, we also searched for cases of *center-embedding*, which would indicate that the complexity of a grammar generating the set of Pirahã sentences is at least context-free.

A major challenge in this analysis is to distinguish embedding from *juxtaposition*. Similar issues have arisen in the analysis of embedding in other languages, such as Warlpiri [41]. Two adjacent sentences might be related to each other semantically, but with no syntactic relationship obtaining between them, in the sense that a computational device which generates the set of grammatical sentences does not require that the two sentences be generated together as part of a single derivation. For example, consider the English examples in (1):

(1a) John went to jail because he drove drunk.
(1b) John drove drunk. That’s why he went to jail.

Examples such as (1a) provide evidence for syntactic embedding. The fragment *Because he drove drunk* cannot appear as a grammatical stand-alone sentence in English, nor can the fragment *John went to jail because*; therefore the sentence cannot be analyzed as two adjacent sentences. The special marker *because* cannot occur in a sentence without another, related sentence present; when we observe a marker with this property, we take it as evidence for embedded sentences.

On the other hand, examples such as (1b) do not provide evidence of syntactic embedding, because both *John drove drunk* and *That’s why he went to jail* can appear as standalone sentences, although there is clearly an inferrable discourse relationship between them. The words *that’s why* often appear after some preceding sentence, but they do not have to. The latter sentence could appear without a preceding sentence if discourse context makes it clear what content the word *that* refers to: for example, one could point to a photograph of John robbing a bank and say *That’s why John went to jail*. The discourse relationship between sentences as in (1b) is not explicit in the syntax, but is rather inferred by the listener in the course of interpreting the pronoun *that*. If we see that discourse markers such as *that’s why* can appear alone, without a preceding sentence, then we do not take them as evidence for embedded sentences.

Within cases of syntactic embedding, cases of *center-embedding* are of particular interest, because if the grammar of a language allows such structures productively, then the language cannot be regular; it must be context-free or more complex. Center embedding consists of cases such as the example in (2):

(2) Because [John drove drunk], he went to jail.

In this case, the presence of the word *because* followed by a sentence (in this case *John drove drunk*) requires the presence of a second sentence, in this case *he went to jail*. An automaton producing words from left to right would have to keep this requirement in memory (on a stack) during the production of the embedded sentence *John drove drunk*. If this embedding can be iterated without bound, then the automaton requires unbounded memory, and so cannot be a finite-state machine; in that case the language generated cannot be regular. Production of these kinds of sentences requires at least a pushdown automaton, corresponding to a context-free grammar [13].

Our approach was therefore to look for cases where discourse structure and the meanings of the sentence lead us to suspect that there might be embedding rather than juxtaposition. We then tried to determine if there was any syntactic characteristic, such as complementizers or word order patterns, that distinguished these from cases of clear juxtaposition. Such a syntactic characteristic would indicate a dependency between one sentence and another, which could be analyzed as an embedding relation.

Using this approach, we examined the following phenomena in the corpus:

### Embedded possessives

One possible form of recursive embedding is embedded possessives, as in English (((*the woman*)*’s sister*)*’s husband*). The presence of this doubly-possessed structure suggests that the grammar contains a rule whereby NPs can contain other NPs, which themselves can contain other NPs, and so on, thus generating an unbounded number of sentences. On the other hand, a language might only allow a single possessor, such as (*the woman’s sister*); in which case we would analyze the possessor phrase (*the woman’s*) as a separate category from NPs. If we observe only single possessors in the corpus, then this analysis is reasonable.

### Reported speech

A particularly common form of embedding in language is reported speech, in which a sentence contains reports of other sentences. This can take the form of direct quotations, e.g. *He said “I’m going”*, or indirect quotations, e.g. *He said that he was going*. Both of these forms can provide evidence for context-free structure, in the form of sentences such as the English *He said “I am going” loudly*, in which there is a dependency of potentially unbounded length between material on both sides of the embedded material *“I am going”*.

Indirect reported speech can provide evidence for recursive embedding, in the form of sentences such as *He said [that she said [that …]]* where the embedding can be iterated without bound. On the other hand, it is unclear whether *direct* reported speech can provide evidence for recursive embedding. A sentence such as *He said “she said ‘Bob said …’”* appears naïvely to contain embedding that can be iterated without bound. However, the quoted material need not be sentence: it could be multiple sentences, or sentences in another languages, or *ungrammatical* sentences as in a linguistics paper, or meaningless sounds like animal noises (e.g., *The owl said “Hoot hoot”*). If we consider the set of sentences in a language to contain the full space of possible direct speech reports, then we would have to conclude that any language with direct quotations is trivially infinite, because one could embed sequences of sounds of unbounded length as quotations. In general it seems odd to include the contents of direct speech reports as part of a language, because they are just reports of the words—or even just the sounds—that someone else said.

Nevertheless, a psycholinguistic case could be made for including a subset of direct speech reports as sentences in a language, at least from the perspective of human sentence production. Most quotations in speech are paraphrases and are likely generated by the same or similar psychological processes as normal sentences [42, 43]. If quoted material is embedded in the sentence that introduces it, then one could argue that the psychological process that generates the quoted material is of the same type as the process generating the containing sentence, so there is recursion in a psychological sense. It is not clear whether this argument for recursion in the human sentence generation process is relevant for characterizing Pirahã with a formal grammar.

For these reasons, although we will discuss cases of direct speech reports in the corpus, we do not take them to be cases of recursive embedding in terms of formal grammar. We will discuss the question of whether speech reports, whether direct or indirect, can be said to be contained in the sentence that introduces it. This is a prerequisite for any possible argument for recursive embedding based on reported speech.

### Sentential complements

These are cases where a sentence is embedded within another sentence as a complement of a verb. For example, in the English sentence *I dreamed that the Brazilian woman was there last night*, the sentence *the Brazilian woman was there* is embedded as the complement of the verb *dreamed*. Depending on the desired meaning, the phrase *last night* can modify the top-level verb *dreamed* (if the dreaming took place last night) or the embedded clause (if the dream was that the woman was there last night). In the former case, this is center embedding and thus evidence for context-free structure. In the latter case, the complementizer *that* would provide evidence of recursive embedding if it were found to mark such cases distinctively.

### Adverbials

Another common locus for sentences embedded within sentences is in content-clause complements of lexical heads, such as adverbials, such as *Because* *S*, *S*.

### Relative clauses

Relative clauses, such as the clause *that the man devoured* in the noun phrase *the food that the man devoured*, are an instance of a sentence embedded in a noun phrase (which may in turn be embedded in a sentence). A dependency may exist between the form of the enclosing noun phrase and the form of the embedded sentence. For example, in English, *the man devoured* is not a complete sentence, but it does qualify as a complete clause when embedded as a relative clause whose head noun can serve as the object of *devoured* such as *food*. This dependency between a clause and a constituent outside it (in this case between *the man devoured* and *food*) is one way in which embedding can be marked. We examined the Pirahã corpus for evidence of relative clauses and for these kinds of dependencies between the embedded clause and their containing noun phrase.

### Complementizers

We examined the corpus for evidence of complementizers, syntactic elements that mark embedded sentences. For example, in English sentences such as *I dreamed that* *S*, the word *that* indicates that the following element is a sentence.

### Coordination

In English, an NP can be extended to arbitrary length by coordination: that is, conjunction (e.g., *John and Mary and Bill and …*) or disjunction (e.g., *John or Mary or Bill or …*). If such a structure does not have an apparent upper bound on the number of conjoined/disjoined nouns, then it provides evidence that the set of sentences is infinite.

## An Analysis of Embedding in the Corpus

In this section we draw attention to what we believe are the pivotal examples in the corpus which might be construed as having embedding. We discuss the issues that arise in the analysis of each kind of example. In the course of this analysis, we consulted highly proficient L2 speakers of Pirahã: Dan Everett, Steve Sheldon, and Keren Madora. Here we present representative examples and analyze them in detail. In most of the cases below, we report the “best” example from the corpus, meaning the one which is closest to exhibiting the phenomenon of interest.

Due to the challenges in parsing and analyzing the language, all our findings here are tentative and should be taken as directions for further study.

Since this is a corpus study and we do not have access to native speaker intuitions, we have to search for structures which are *unambiguously* instances of embedding. Such unambiguously embedded structures are rare in natural speech even in languages which certainly have them [44]. They are even rarer in polysynthetic languages [45], of which Pirahã is one. This consideration raises the possibility that such structures might be possible in Pirahã, but not attested in this corpus.

In the analysis below, for the structures we attempt to identify in the Pirahã corpus, we also give the frequency of those structures in the Switchboard corpus of English phone conversations [46]. The aim is to give a rough sense of whether the presence of absence of some structure in the Pirahã corpus is informative; i.e., if a structure is extremely rare in English, it might be rare in other languages also, and then maybe it is not informative that we do not find it in our small Pirahã corpus. However, due to the large cultural differences between the Pirahã and English speakers, and between the settings in which these corpora were collected, this comparison is rough.

In this section, when we give interlinear glosses, we mark clitics in the following way: the first-person clitic *ti* is 1, the generic third-person clitic *hi* or *k* is 3, the third-person clitic for foreigners *ao* is foreigner, and the third-person clitic for animals *ísi* is animal. cont stands for continuative; inter stands for interrogative;

### Embedded Possessives

Possessors appear in Pirahã syntax as either a clitic or a full nominal expression before the possessum, as in sentences 11.14.3 and 5.27.1 respectively

11.14.3

 hi giopaí oó xiai

 3 dog jungle be

 “His dog is in the jungle.”

5.27.1

 ahoógi hoí k oaí-koí tiobáhai

 A. son 3 die-emphatic child

 “Ahoógi’s child died!”

We find many such possessors, but no unambiguous instances of nesting within possessors. For comparison, nested possessors appear in the English Switchboard corpus at a rate of 1.6 per 1000 sentences.So it would be no surprise if this syntactic structure existed in Pirahã, but we failed to find an example in this corpus of 1149 short sentences. The only potential example we find in the corpus is sentence 1.60.

1.60.1

 Xoii hi aigía hi áhaig-ó kagi otí ∅-haí

 X. 3 thus 3 sibling-direction companion angry (NullVerb)-certainty

 “Xoii was thus really mad at his brother’s wife (it seems).”

The structural analysis of 1.60.1 hinges on the status of the second clitic *hi*. In an analysis with recursive embedding (as in (3a)) it could be a possessive attached to the following word *áhaig-ó*, but in an analysis without embedding (as in (3b)), it could be a repeated subject clitic. (This is the only instance of a possessor marked with *-ó* in the corpus; *-ó* is usually a directional marker.) As we will show, repeated subject markers are common elsewhere in the corpus.

(3a) (((hi) áhaig-ó) kagi)
(3b) (hi) ((áhaig-ó) kagi)

To get a sense of the likelihood of the second analysis, we should establish whether *áhaig* by itself (i.e. not preceded by *hi*) can mean “his brother”. *áhaig* appears to have the meaning “(my/his/her) sibling” in several sentences (e.g., 1.39.3, 9.38.1, 10.57.1). Out of 19 occurrences of *áhaig* meaning “sibling” (excluding 1.60.1), there are only two instances where it is preceded by any NP with a possible possessive reading. So *áhaig* usually appears alone and can mean “his sibling”: this suggests the analysis of the observed clitic *hi* in sentence 1.60.1 as a repetition of the subject rather than a possessive.

We should also determine how often *hi* is a repeated subject clitic as opposed to a possessor clitic in the position NPsubj *hi* *aigía* *hi*. We findfour instances of repeated subject clitic *hi* in this context; for example, sentence 12.23.1:

12.23.1

 kabógo hi aigía hi ab-ií

 K. 3 thus 3 remain-intent

 “Kabógo intends to stay [here]”

More generally, we can look at the distribution of NPs following *NPsubj* *aigía*. We finda basically uniform distribution of subjects (25/49) and objects (24/49) in this position. The high frequency of repeated subjects in this context supports the plausibility of reading sentence 10.60.1 as containing a repeated subject, as opposed to a nested possessive.

### Reported speech

Here we discuss utterances of the form “*NP* said *X*”, where *X* is a direct or indirect report of speech. In order to facilitate the study of these phenomena, we have provisionally labeled sentences glossed as direct quotations with the category *Q* in the corpus. Indirect quotations are rare in the corpus, and also hard to detect, because there is no obvious complementizer (as we discuss below) and because the clitic *hi* can be used for the first or third person. This ambiguity means that sentences such as *He says ‘I’m leaving”* and *He says he’s leaving* are indistinguishable. For this reason, we do not attempt an analysis distinguishing between the speech reports glossed as direct and indirect; it is possible that they are all direct.

Reported speech in Pirahã typically appears in the form “*NP* said *X*” and so it is possible to analyze these utterances syntactically as separate sentences, “*NP* spoke” followed by the contents of the reported speech *X*. There are also a few instances of the form “*X*, *NP* said” (e.g., sentence 10.84).

Pirahã sentences are typically verb-final except for some topicalized noun phrases appearing after the verb. With this in mind, if the reported speech *X* were syntactically the object of the verb *said*, a natural place for it to appear would be before the verb. Nonetheless many languages (such as Turkish) treat sentential objects and reported speech in a syntactically special way, so it would not be surprising if Pirahã made an exception to its verb-finality for reported speech.

We looked for center-embedding of quotations, in which a syntactic dependency exists between material on two sides of some reported speech. We found no such examples. Such examples are also rare in English conversation: the rate in Switchboard is 1.3 per 10,000 sentences.So such a structure might be possible in Pirahã but not present in the corpus.

We also considered the frequency of occurrence of reported speech after the common verbs for “speak”. If the verbs for “speak” were *always* followed by a speech report *X*, then that would be evidence that the report *X* is embedded in the sentence containing “speak”, perhaps as an object or complement of the verb. On the other hand, if the verbs appear without any following reported speech, then that finding would support their possible reading as intransitive verbs.

Reported speech is almost always introduced by a variant of the verb *gá*; Sentence 5.17 is a typical example:

5.17 Original gloss: Oi then said, I’m not going away from the girls.

5.17.1

 Xoi hi aigía gá-xai

 X. 3 thus speak-do

 “Xoi thus spoke.”

5.17.2

 ∅ aogi aíso xai-kab-i-haí

 NullSubject foreign.woman also do-neg-(epenthetic)-relative.certainty

 “[I] also will not [leave] the foreign woman.”

The verb *gá* was originally glossed as “say”, potentially taking a sentential complement, but in Dan Everett’s more recent analysis it is glossed as intransitive “speak” or “carry sound”[47]. In the vast majority of instances in our corpus, *gá* is followed by reported speech. There is another verb *ahoa*, glossed as “talk”, which is usually used to describe talking where no reported speech is given, as in 10.63.1:

10.63.1

 ∅ áhaig áhoa-hoagaí-ihí

 NullSubject sister talk-come-inter

 “Did [she] come to talk [to her] sister?”

The existence of the two verbs raises the possibility that *gá* is a transitive verb like English “say”, taking the reported speech as a complement, while *ahoa* is an intransitive verb like English “talk”. However, in Sentence 9.50, *gá* is attested without a quotation, and *ahoa* is attested introducing one. This supports the analysis of *gá* and *ahoa* as intransitive verbs meaning “talk”, where both might be followed by reported speech.

9.50 Original gloss: Should I go up and talk to them?

9.50.1

 hi ahoa

 3 talk

 “He talked.”

9.50.2

 ∅ igá-boí-xiig-oxoi-hí-i-sai-híaha

 NullSubject speak-cause-cont-inter-inter-?-oldinfo-hearsay

 “[Should I] continue to speak [to them] (as I have heard and has been mentioned)?”

While *gá* and *ahoa* are both attested with and without reported speech, the fact remains that, numerically, *gá* is almost always followed by reported speech (183/191 cases), while *ahoa* rarely is (2/20 cases). If we take a classical approach to syntax, aiming to model only the distinction between grammatical sentences and ungrammatical ones without regard to relative frequencies, then this data is irrelevant. In that case, the high frequency of reported speech after *gá* must be considered an accident or a result of the semantics of *gá*.

On the other hand, if we wish to model Pirahã with a probabilistic generative model, as is standard in computational linguistics [48] and psycholinguistics [49, 50], then it might be favorable to analyze the reported speech as embedded in the sentence that introduces it. The distribution of reported speech after *gá* could be modeled by having a high-probability rule such as *VP*→ gáisai *Q* in addition to a low-probability rule such as *VP*→ gáisai. We leave it to future work to determine whether such a grammar would provide a better fit to the corpus data.

In summary, there is no evidence of center-embedding of reported speech, and it is not clear whether reported speech can said to be contained within the sentences that introduce them. The corpus is consistent with both analyses: one where speech reports form separate sentences, and one where speech reports are embedded after the verbs *gá* and (rarely) *ahoa*.

### Discontinuous Quotations

In addition to sentences preceded by *gá* and *ahoa*, we also find two examples where these verbs intervening in the course of a quotation. The result might be analyzed as a discontinuous constituent, a hallmark of non-context-free structure. Examples are found in sentences 13.12 and 14.13.

13.21 Original gloss: Steve said, “Tomorrow I will look for bananas.”

13.12.1

 poogaíhiai

 bananas

 “Bananas.”

13.12.2

 ao gá-sai-híai

 foreigner speak-oldinfo-hearsay

 “(I heard that as has been mentioned,) the foreigner [Steve] spoke.”

13.12.3

 ahoahi-ó ao haoxá-isai-híai

 tomorrow-loc foreigner search-oldinfo-hearsay

 “(I heard that as has been mentioned,) tomorrow [I] the foreigner will search.”

This can be interpreted as the single sentence: *“Bananas,” he said, “I will search for them.”* Even if this is the correct analysis of the utterance, the syntactic implications of this structure are unclear even in English; McCawley [51] has argued that the verb *say* in such parentheticals is either intransitive or takes a null object. Under that analysis, the English sentence could be analyzed as three independent sentences.

### Sentential complements

Sentences can be embedded in other sentences in ways other than as reported speech; here we examine the case of sentential complements, where sentences function as arguments of verbs.

Null subjects and ambiguous clitics obscure our ability to discern embedded sentences. For example, Sentence 8.2 below could be interpreted as “I started dreaming that the foreign woman was there”, in the order “I [that the foreign woman was there] started dreaming”. On the other hand, the initial clitic *ti* could be interpreted as a topic, meaning “with respect to me”, with the first sentence meaning “With respect to me, the foreign woman was there”, an independent sentence. This interpretation holds that the clitic is similar to the “ethical dative” in Romance languages [52].

8.2 Original gloss: As I dreamed, I and the Brazilian woman were there.

8.2.1

 ti xaí aogí ai-xaagá

 1 thus foreign.woman do-be

 “Well, [with respect to] me [my dream], the foreign woman was there.”

8.2.2

 ∅ apipaó-ba-hoagaí

 (NullSubject) dream-durative-inchoative

 “[I] began dreaming.”

If we analyze 8.2.2 as a separate sentence, it has a null subject; but we could also analyze 8.2.1 and 8.2.2 as one sentence, where the content of 8.2.1 is the object of the verb in 8.2.2. This would fit the OV structure of the language. However, the plausibility of a null-subject analysis is increased by the fact that null subjects are very common in general in the corpus, making up 22% of subjects according to our parses, for the most part in cases where it is implausible that a previous sentence is the missing subject.

In summary, we do not find unambiguous evidence that sentences can be embedded as arguments of verbs in Pirahã. So we cannot conclude the existence of recursive embedding or infinitude on the basis of sentential complements.

### Adverbials

In adverbials, a sentence is embedded into another sentence as an clausal complement of a preposition such as *after*. The resulting phrase modifies the verb. For example, the English sentence *After John arrived, the party began* contains an adverbial (underlined). If we can find evidence of a syntactic relation between a sentence and an apparent adverbial clause, then this would provide evidence that these form a higher syntactic unit, suggesting recursive embedding and sentences of unbounded length. We find some sentences that might be interpreted as containing adverbials, which include a morpheme *-aó* that might be a complementizer meaning “when”. For example, we might interpret sentence 12.40 as having an embedded adverbial sentence (12.40.1) marked with the *-aó*.

12.40 Original gloss: When he went to look for body paint, he found the tracks.

12.40.1

 ∅ aixií aog-i ap-aó

 (NullSubject) annatto look-? go-completive

 “[He] went to look for annatto.”

12.40.2

 hi ísaó apoí aihíop-aí

 3 tracks on.top.of find-do

 “He found/came upon the tracks.”

In the corpus we have 34 examples of *-aó* annotated as a completive morpheme at the end of a verb. Of those, 24 appear in contexts where they could be interpreted as complementizers marking an embedded sentence. However, in the remaining 10 examples, *-aó* appears at the end of a bare sentence, or it appears without an adjacent sentence into which it could be sensibly embedded (Sentences 1.26.1, 1.49.2, 1.56.3, 1.59.6, 1.65.3, 1.73.4, 1.79.2, 1.96.2, 1.98.2, and 1.101.1).

We find some fixed adverbial temporal phrases which could be analyzed as containing embedded sentences, but the apparent embedding is highly unlikely to be productive. Specifically, the expressions meaning “at dawn” and “at night” can be broken down as compounds of nouns and verbs, suggesting possible embedding. For example, the word for “dawn” in Dan Everett’s analysis is literally “day-eat-night” (*ahoa-kooho-ahío*) in sentences 5.36.1, 5.38.1, 5.42.1, 9.27.1, and 9.51.2; and the word for “night” is literally “cause-fire (to be lit)” (*xa-hoa*) in sentence 4.20.1. However, these fixed phrases, which repeat in almost identical forms in each instance, are likely best analyzed as individual lexical items, rather than sites of productive embedding.

Thus, in summary, we do not find strong evidence of a syntactic relation between sentences and apparent adverbials modifying them, so we cannot determine if recursive embedding is present on the basis of adverbials in the corpus.

### Relative Clauses

A relative clause is a sentence embedded in an NP; this provides another possible locus of embedding and hence the possibility to generate an infinite number of sentences. Relative clauses also have the potential to give rise to center-embedding, which would provide evidence for context-free structure in Pirahã.

We find one pair of sentences that might be analyzed as one sentence containing a relative clause. This is sentence 9.3, which could be interpreted as “a group that is not small can carry her up the riverbank.”

9.3 Original gloss: Only one cannot carry her up the river bank.

9.3.1

 hi hoi-hiab áa-há

 3 few-neg be-complete.certainty

 “Certainly, they are not one [person].”

9.3.2

 ∅ iig-op-ai-saí

 (NullSubject) carry-go-be-oldinfo

 “(As has been mentioned,) [they] carry [Xaogioso up the river bank].”

Like the analysis of the possible sentential object in sentence 8.2 above, the analysis here depends on the likelihood that the second sentence contains a null subject, as opposed to having the first sentence as an object. The plausibility of the null-subject analysis is increased by the high frequency of null subjects throughout the corpus in other contexts. Thus the relative clause analysis is less likely.

Since we do not find unambiguous relative clauses in the corpus, we cannot use them to conclude that Pirahã has recursive embedding.

### Nominalizers and Complementizers

Here we discuss cases where a sentence which might be embedded into another sentence as an NP carries some syntactic or morphological marking which marks it as embedded. Such marking would provide evidence for a syntactic relationship between the sentence and the putatively embedded sentence, and thus recursive embedding. We focus on potential complementizer morphemes that have been discussed in previous literature, or which we discovered in the course of our analysis of the corpus.

#### *-si* Nominalizer/Complementizer

The suffix *-si* was originally glossed as a nominalizer or complementizer. For example, sentence 1.66 can be interpreted as “That he is not ignorant is certain.” On the other hand, the negative morpheme *xaab* might mean something like “that is false”, which would not require *syntactic* embedding.

1.66 Original gloss: He is ignorant. He is not.

1.66.1

 hi o-s ai-si

 3 eye-neg be-nominalizer?

 “He is ignorant.”

1.66.2

 ∅ xaab á-há

 (NullSubject) neg be-complete.certainty

 “Certainly not.”

The suffix *-si* is amply attested in our corpus as a suffix on adjectives and adverbs which serve as nouns: 12/23 appearances of *-si* are in this context. If *-si* is a nominalizer for adjectives and adverbs, it could also easily be a nominalizer for embedded sentences or verb phrases. However, if it is a nominalizer, it is far from obligatory: only 8/106 adjectives serving as object NPs are marked with *-si*. The optionality of *-si*, along with its frequent presence outside of a nominalization context, suggest that it has some other meaning, such as marking old information, rather than being a marker of nominalization.

#### *-saí* Complementizer

Sauerland [53] claims that the morpheme *-saí* (with high tone), as opposed to *-sai* (with low tone), functions as a complementizer (cf. [54]). We find 262 instances of *-sai* and 9 instances of *-saí* in the corpus; both are usually glossed as an old-information marker. They appear in many contexts such as 5.35.1 and 12.17.1 where there is no plausible embedded clause.

5.35 Original gloss: He lives near the girls.

5.35.1

 hi aógi hi xia-haxa-isai-hiái

 3 foreign.woman 3 live-complete.certainty-oldinfo-hearsay

 “(I heard that as has been mentioned,) he certainly lives [near] the foreign women.”

12.17 Original gloss: She will eat the head of the tapir.

12.17.1

 ∅ apa óhoi-haí kabatií-ísaí

 (NullSubject) head eat-relative.certainty tapir-oldinfo

 “(As has been mentioned,) [she will] eat the head [of the] tapir.”

The appearance of these morphemes in positions where embedding is implausible suggests that they do not mark embedding, but rather have some other meaning; then when they appear in apparent embedding contexts, the simplest analysis is that they are expressing that meaning rather than marking embedding.

To summarize, we do not find strong evidence for morphemes that mark embedded sentences, which calls into question whether any example of a potentially embedded sentence really is embedded rather than just placed adjacent to another sentence. The potential complementizers that we identify could easily be markers of other properties of the words they modify, such as their discourse status.

### Coordination

Coordination, in the form of conjunction and disjunction, is a construction that can potentially be iterated without bound, though its presence does not imply context-free recursive structure. We searched for instances in the corpus where the English translation includes “and” or “or”. We found no examples of disjunction in the corpus. By comparison, NPs coordinated with “or” occur at a rate of 1.1 per 100 sentences in the Switchboard corpus.

We found five examples where NPs within sentences might be conjoined (8.2, 8.5, 10.43, 12.3, 12.52). By comparison, the Switchboard corpus has 2.7 conjoined NPs per 100 sentences.The large majority of sentences whose English translation includes the word *and* mean it in the sense of “and then”, appearing at the beginnings of sentences. In 3 out of 5 cases, the NPs which appear to be conjoined semantically are expressed simply by including both NPs as subjects without any special syntactic or morphological marking for conjunction. In the remaining cases, sentences 12.3 and 12.52, the second apparent conjunct is followed by *pío*. This could potentially be a syntactic marker of conjunction, but it appears elsewhere in the corpus as an adverb with the meaning “also” in sentences containing only one NP (e.g. 15.6).

The apparently conjoined NPs may be separated by discourse markers such as *xaigía* “thus”, so it is not clear that they form a single compound NP constituent. However, even if the semantically conjoined NPs do not form a single syntactic unit, there is possibly no upper bound on the number of semantically conjoined NPs in a single sentence. On the other hand, even if these are properly analyzed as conjuncts, then it is possible that no more than two are permitted per sentence: we find no examples of apparent conjunction of more than two noun phrases.

In Everett’s analysis and translation, which we follow in our parse of the corpus, these are not instances of conjunction semantically. The possibility of conjunction is based on Steve Sheldon’s original glosses. In Everett’s analysis, one of the apparently conjoined NPs is analyzed as a topic placed at the beginning of the sentence. Under the topic analysis, the construction cannot be iterated, and so does not lead to sentences of unbounded length.

In favor of the topic analysis, we note that in Sentence 8.5 (describing a dream), it is felicitous in context to interpret the sentence as “the Brazilian woman disappeared”, without the speaker also disappearing. It would be odd for a speaker to say that he himself disappeared.

The non-conjunct reading also makes sense in context for Sentence 12.3. The previous sentence had mentioned that the speaker Xoi was fishing, so it is plausible that the clitic *hi* referring to Xoi marks the continuation of a topic rather than a conjunction. According to Dan Everett’s analysis, with Steve Sheldon concurring, the second NP (meaning “his relative”) is an appositive, coreferential with the first NP in this sentence. (On the other hand, Keren Madora interprets the sentence as “Toiao and his brother will keep eating baiosi (contrary to expectations)”.) The presence of the discourse particle *aigía* might also indicate that the clitics in question as topical here, although *aigía* appears frequently after NPs glossed as subjects elsewhere throughout the corpus.

8.5 Original gloss: “Well, then I and the big Brazilian woman disappeared.”

8.5.1

 ti xaigía ao ogí gió ai hi ah-á-p-i-ta

 1 thus foreigner big much thus 3 go-vertical-up-(epenthetic)-repetitive

 “Well, [with respect to] me, the very big foreigner went away again.”

12.3 Original gloss: “He and his relatives were fishing for piranha.”

12.3.1

 hi aigía ahai pío ísi hoa-ab-i-sai-híai

 3 thus sibling also animal search-durative-(epenthetic)-oldinfo-hearsay

 “(I heard that as has been mentioned,) thus he [Xoi], his relative, is also searching for animals.”

In summary, the corpus does not provide unambiguous evidence for coordination structures. There is possible evidence for coordination in the form of lists of subjects without any explicit word for “and” or “or”, but even if this analysis is true, it is not clear that this coordination can be iterated.

## Discussion

We found no unambiguous evidence for sentential or NP embedding in Pirahã in our corpus. The corpus is consistent with the hypothesis that Pirahã is a regular language; we leave it to future work to determine whether this is the *best* analysis, considering all the possible analyses of the examples discussed above.

In order to flesh out our claim that the corpus is consistent with a regular grammar, we give here a regular expression (technically an egrep expression) which is consistent with the corpus. The symbol S matches all sentences in the corpus:

S = NPtopic? NPtopic? NPvoc? NPsubj NPsubj? NPsubj? NPtmp? NPloc? NPiobj? (JJobj | NPobj NPobj?)? NPiobj? V JJobj? NPvoc? NPtopic?

where *X*? means optional *X*, (*X*|*Y*) means *X* or *Y*, and each of the symbols above expand into other regular expressions (ignoring morphology and null nouns/verbs):

NPsubj = (NNP | JJ? PRP | PRP DT? | POS? POS? NN | POS? NN DT | POS RB NN | JJ PRP? | JJR | POS? JJ N | NN DT? JJ | NN? WP)
NPobj = (PRP | POS? POS? NN | NN (DT | JJ) | POS JJ)
NPiobj = (PRP | POS? POS? NN | NN JJ)
NPtopic = (NNP | PRP CD? | POS? NN | NN JJ)
NPloc = (NNP | PRP | NN (DT | JJ | CD)? | DT NN | CD | JJ)
NPtmp = NN JJ?
NPvoc = NNP
POS = (NNP | PRP | NN | JJ)
JJobj = (JJ DT? | CD | DT)
PP = NPobj IN

Also, in the expression for S above, we should allow intervening prepositional phrases and adverbs; this is equivalent to writing (PP? RB? | RB? PP?) between each symbol. This expression does not have any symbol expanding transitively to itself, so it does not match an infinite number of strings. This description encompasses many sentences which might include false starts and parentheticals, so it is possible that an even simpler regular expression for Pirahã is possible.

The regular expressions above can be distilled down to a simple description of a Pirahã sentence in terms of linear precedence of phrases: topic* > vocative > subject* > NPtmp* > NPloc* > (indirect object > object* | object* > indirect object) > V > adjectival object > topic, where * means “up to 3 instances”, > means “linearly precedes”, and (*X*|*Y*) means *X* or *Y*. Any of the *X* *s in this expression could instead be interpreted as allowing unbounded repetitions of *X*. If we interpret * as allowing unbounded repetition, then we analyze Pirahã as an infinite regular language.

We do not claim that this grammar is the best grammar for the corpus; it might be that a simpler grammar would have recursive embedding, for some definition of “simple”. We hope that the data we are currently releasing will make it possible to find the best grammar. Currently, we only claim that the grammar above is consistent with the corpus.

## Conclusion

Our analysis has failed to find strong support for syntactically embedded structures in Pirahã. We emphasize that any conclusions that can be drawn from this corpus evidence must be highly tentative, due to the difficulty of working with a language whose speakers are so difficult to access, as well as the computational challenges of characterizing linguistic complexity. Our hope is that the analysis presented here, along with the release of the annotated corpus, will promote further investigation into the formal properties of natural languages and help to push the debate towards testable empirical claims.
